# Medical Liberalism

**Published:** 1895-08

**Authors:** 


### Abstracts and Selections.

### Medical Liberalism.

Whether in politics, religion or science there is no word around the talismanic influence of which there gathers such a halo of speciousness as that one word—*liberalism*. In its highest and noblest sense no man, above all, no true physician, can afford to be anything else but liberal, but there is a liberalism which we shall define only to condemn and which to an alarming extent is poisoning the pulses of the body medical—a liberalism the sophistry of which has led many men of noble instincts and inborn purity of principle into the condoning of evil practices and even into collusion with the base methods of the empiric and the charlatan. It is time for those upon the outlooks of the profession to sound the danger signal before this Trojan horse is wheeled within our gates to the destruction of our time-honored ethical principles and the everlasting ruin of the shrines and monuments of professional honor.

It is in our great cities where this cry of liberalism is loudest sounded and produces most disastrous effects upon the organon of professional life. In the city of New York—the great metropolis which ought to be without spot or blemish—the guide of professional life, the paragon of professional honor—it is humiliating to see how this spirit of liberalism, falsely so-called, has carried the very highest and noblest and most renowned of medical men. Thé wealthy and ultra fashionable elements of society—the 140—have decided among themselves that without regard to principle or theory or the demonstration of fact the ordinary ailments which come and go as clouds float by in the heavens, are more pleasantly treated by the refined estheticism of therapeutics, generally known as homeopathy, and to explode the fallacies of its philosophy means dismissal from service in those conditions where skill, intelligence and scientific medicine are in great demand. The public are ignorant of the principles upon which all true medical philosophy is founded. To them a doctor is a doctor and they do not stop to inquire into the reasons which forbid the conscientions physician from conniving at charlatanry and imposture, and if he hesitates he is lost, for at once goes up the shout, "He is *illiberal*," "He is afraid to meet the 'members of the other school,' " as they style themselves, borrowing the dignity of medicine and thus hanging the tattered shreds of their charlatanry upon the colossal columns of our glorious calling. Men are afraid to say their souls are their own, lest they should be laughed at as "old fogies," as "non-progressive," as unfashionable—as "*illiberal!*" And it is this which has broken up forever in our great cities that once holy relation of the physician to the family, when he was the chief of all the household gods—the oracle of the community in which he lived. All this is gone forever, and with it, too, has gone that salutary influence upon social life, that sincere reverence for truth at whatever cost, that devotion to principle, that self-denying consecration to duty, that lofty sense of professonal honor which made a "doctor" in the eyes of men "but a little lower than the angels." Let us come back to our first love, let us reform it and reform it altogether. *O Jerusalem, Jerusalem, convertite ad Dominum!"--Ed. St. Louis Clinique.*

